# A Nomogram Based on the Castelli Risk Index for Predicting Prognosis in Patients With Acute Coronary Syndrome Undergoing Percutaneous Coronary Intervention: A Cohort Study

**DOI:** 10.1002/iid3.70431

**Published:** 2026-04-07

**Authors:** Yan Liu, Ge Song, Ying Zhang, Weichao Shan, Yuewen Qi, Xinchen Wang, Chen Wei, Jingyi Liu, Lixian Sun

**Affiliations:** ^1^ Department of Cardiology The Affiliated Hospital of Chengde Medical University Chengde China; ^2^ The Cardiovascular Research Institute of Chengde Chengde China; ^3^ Hebei Key Laboratory of Panvascular Diseases Chengde China; ^4^ Central Laboratory of Chengde Medical University Affiliated Hospital Chengde China

**Keywords:** composite lipid indices, dyslipidemia, high‐density lipoprotein cholesterol, low‐density lipoprotein cholesterol, major adverse cardiovascular events

## Abstract

**Background and Aims:**

Composite lipid indices are closely related to the risk and poor prognosis of diseases. However, there are no studies on the prognostic value of Castelli's risk indices‐I and II (CRI‐I, CRI‐II) in patients with acute coronary syndrome (ACS) undergoing percutaneous coronary intervention (PCI). Therefore, the aim of this study was to investigate the association of CRI‐I and CRI‐II with the prognosis of patients with ACS undergoing PCI.

**Patients and Methods:**

A total of 1475 patients with ACS undergoing PCI were consecutively enrolled in this prospective cohort study from January 2016 to December 2018. The CRI‐I and CRI‐II were measured. The endpoints were MACEs, including all‐cause mortality, requirement of rehospitalization for severe heart failure, recurrence of myocardial infarction, in‐stent restenosis, and reaccept PCI. Follow‐up data were collected via clinical visits or telephone calls at 1, 3, 6, and 12 months and annually thereafter.

**Result:**

Multivariable Cox regression analysis revealed that the risk of MACEs increased gradually with increasing CRI‐I and CRI‐II. The cumulative survival rate in the CRI‐I ≥ 3.350 and CRI‐II ≥ 1.697 groups was significantly lower than that in the CRI‐I < 3.350 and CRI‐II < 1.697 groups, respectively (log‐rank tests: all *p* < 0.001). The nomogram demonstrated good predictive performance for the 1‐, 2‐, and 3‐year survival probability.

**Conclusions:**

CRI‐I ≥ 3.350 and CRI‐II ≥ 1.697 could serve as independent predictors of MACEs in patients with ACS undergoing PCI.

## 1 Introduction

Cardiovascular disease (CVD) is the leading cause of death worldwide, with nearly half of these deaths caused by ischemic heart disease [1]. ST‐segment elevation myocardial infarction (STEMI), non‐STEMI (NSTEMI), and unstable angina are the main characteristics of acute coronary syndromes (ACS), and there are more than 7 million people who suffered ACS annually worldwide [2]. Atherosclerosis and plaque disruption are the underlying culprits in patients with ACS [3]. In the past decades, plenty of mechanisms have been researched as the underlying pathogenesis of atherosclerosis, one of the most important is dyslipidemia, which is defined as increased plasma concentrations of total cholesterol (TC), low density lipoprotein cholesterol (LDL‐C), and triglycerides (TG) or decreased plasma concentrations of high‐density lipoprotein cholesterol (HDL‐C), as well as the presence of abnormal lipoproteins that are not routinely measured in clinical practice, such as nitrotyrosine, nitrated lipoproteins [4, 5]. In clinical practice, the TC to HDL‐C ratio has been used as a composite lipid index to evaluate CVD risk scores and the levels of serum thyroid and thyroid‐stimulating hormones [6, 7]. Therefore, we hypothesized that composite lipid indices are capable of offering a more thorough evaluation of a disease compared to individual lipid indices. In recent years, composite lipid indices emerged including the atherogenic index of plasma (AIP), lipoprotein combine index (LCI), cholesterol index (CHOLINDEX), and Castelli's risk indices‑I and II (CRI‐I, CRI‐II). A previous study reported that AIP, LCI, CHOLINDEX, CRI‐I, and CRI‐II are independent predictors of CAD and its severity [8, 9]. Furthermore, AIP and CRI‐II could be used to independently predict coronary slow flow [10]. CRI‐I and CRI‐II, as non‐traditional lipid parameters, are calculated by the following formulas: CRI‐I = TC/HDL‐C and CRI‐II = LDL‐C/HDL‐C [11]. Recent studies have suggested that CRI‐I and CRI‐II are potential predictors of carotid plaque vulnerability and intracranial and extracranial atherosclerotic stenosis [12, 13]. Our team has been investigating the prognostic value of various inflammatory indexes in patients with ACS for several years, including the aggregate index of systemic inflammation (AISI), systemic inflammation response index (SIRI), neutrophil‐to‐lymphocyte*platelet ratio (NLRP), and the advanced lung cancer inflammation index (ALI), and we found that the above markers may be novel indexes for identifying high‐risk patients with ACS after PCI [14, 15]. However, there are no studies on the prognostic value of CRI‐I and CRI‐II in patients with ACS undergoing PCI. Therefore, our study is aim to investigate the relationships of CRI‐I and CRI‐II with the prognosis of patients with ACS undergoing PCI.

## 2 Materials and Methods

### 2.1 Study Population

This study retrospectively analyzed 1475 patients with ACS undergoing PCI from January 2016 to December 2018 at the Affiliated Hospital of Chengde Medical University. The inclusion criteria were as follows: age ≥ 18 years; unstable angina, STEMI, and NSTEMI; coronary arteriography showing stenosis of ≥ 50% in one or more of the left main, left anterior descending, left circumflex, right main, right posterior descending, or right marginal arteries or their main branches; and PCI for the first time. The exclusion criteria were patients with stable angina, severe heart diseases (e.g., aortic dissection and hypertrophic cardiomyopathy), coronary artery spasm, infectious diseases, systemic inflammatory disorders, malignant tumors, blood system diseases, and hepatic and renal dysfunction. Clinical risk factors for typical ACS were determined based on our previous study [16]. This study was approved by the Ethics Committee of the Affiliated Hospital of Chengde Medical University (Number: CYFY LL2015006). All participants provided written informed consent.

### 2.2 Clinical and Laboratory Data Collection

Demographic and clinical characteristics of all patients including typical clinical risk factors for ACS, such as age, hypertension, dyslipidemia, ischemic stroke, diabetes mellitus, history of heart failure (HF), and family history of coronary artery disease, were collected by our team during hospitalization. Blood routine data derived from fasting blood samples, such as TC, TG, HDL‐C, LDL‐C, white blood cell count, platelet count, serum creatinine, and serum uric acid, were also recorded.

### 2.3 Follow‐up and Study Endpoints

The study endpoint was major adverse cardiovascular events (MACEs), including all‐cause mortality, requirement of rehospitalization for severe HF, recurrence of myocardial infarction, in‐stent restenosis, and reaccept PCI. All‐cause mortality was defined as death from any cause. Severe HF was defined as New York Heart Association Classification Class IV. Follow‐up data were collected via electronic medical records and/or clinic visits at 1, 3, 6, and 12 months and once a year thereafter. All researchers were trained using the same criteria and protocols.

### 2.4 Statistical Analyses

Statistical analyses were performed using SPSS 26.0 (SPSS Inc, Chicago, IL, USA), GraphPad Prism 8.0 (GraphPad Software Inc., La Jolla., CA), and R 4.3.3 software. The missing data can be handled automated by setting the exclusion of both user‐missing values and system‐missing values in software. Continuous variables with skewed distribution according to the Kolmogorov–Smirnov test were presented as medians (interquartile ranges), and all categorical variables were presented as frequencies (percentages). The Mann–Whitney *U* test was used to compare the differences between the MACEs and non‐MACEs groups. The *χ* ^2^‐test was used to compare the categorical variables between the two groups. Receiver operating characteristic (ROC) curves were drawn to identify a cutoff value for CRI‐I and CRI‐II according to Youden's index (sensitivity + specificity − 1). Univariate and multivariate Cox proportional hazards models were used to predict the independent risk factors for MACEs. To assess the prognostic performance of CRI‐I and CRI‐II, these indices were assigned to three groups (T1, T2, and T3) according to the tertiles. Survival curves were depicted using the Kaplan–Meier method, and survival rates were compared using the log‐rank test. A restricted cubic spline (RCS) was plotted via the R package functions, “rms” and “ggplot2.” A nomogram was established based on the multivariate Cox analysis results using the R package function, “rms.” The predictive accuracy of the model was determined by a calibration curve, which was plotted using the R package tools, “survival” and “rms.” *p* values < 0.05 were considered statistically significant.

## 3 Results

### 3.1 Baseline Clinical Characteristics

After excluding patients with stable angina, malignant tumors, infectious diseases, and blood system diseases and those lost to follow‐up, our study population included 1475 patients with ACS undergoing PCI. The medium follow‐up period was 1003 days. Table 1 shows the basic clinical characteristics of patients in the MACEs (*n* = 146) and non‐MACEs (*n* = 1329) groups. The proportions of patients with a history of HF, cardiogenic shock, CRI‐I ≥ 3.350, and CRI‐II ≥ 1.697 were significantly higher in the MACEs group than in the non‐MACEs group (*p* < 0.001). There were significant differences in age ≥ 65 years, acute myocardial infarction (AMI), unstable angina (UA), dyslipidemia, WBC count (10^9^/L), TC (mmol/L), HDL‐C (mmol/L), LDL ‐C(mmol/L), and serum creatinine ≥ 110 μmol/L (*p* < 0.05) between patients in the two groups. There were no significant differences in hypertension, diabetes mellitus, ischemic stroke, smoking, family history of CAD, serum uric acid (μmol/L), left ventricular end‐diastolic diameter (LVEDD) ≥ 50 mm, and left ventricular ejection fraction (LVEF) ≤ 40% (*p* > 0.05) between the two groups.

**Table 1:** Baseline clinical characteristics of the MACEs and non‐MACEs groups.

Variables	MACEs group (*n* = 146)	Non‐MACEs group (*n* = 1329)	χ^2^/*Z*	*p* value
**Demographic and clinical data**				
Male	102 (69.9%)	999 (75.2%)	1.957	0.162
Age ≥ 65 years	45 (30.8%)	308 (23.2%)	4.225	0.040
AMI	100 (68.5%)	786 (59.1%)	4.795	0.029
UA	46 (31.5%)	543 (40.9%)	4.795	0.029
Dyslipidemia	99 (67.8%)	742 (55.8%)	7.700	0.006
Hypertension	94 (64.4%)	781 (58.8%)	1.720	0.190
Diabetes mellitus	45 (30.8%)	324 (24.4%)	2.991	0.088
Ischemic stroke	26 (17.8%)	185 (13.9%)	1.622	0.203
History of HF	33 (22.6%)	113 (8.5%)	29.325	< 0.001
Smoking	70 (47.9%)	696 (52.4%)	1.032	0.310
Family history of CAD	20 (13.7%)	194 (14.6%)	0.086	0.770
Cardiogenic shock	9 (6.2%)	13 (1.0%)	20.681	< 0.001
**Laboratory data**				
WBC count (10^9^/L)	8.18 (6.87, 11.31)	7.87 (6.31, 10.21)	−2.205	0.027
Platelet count (10^9^/L)	215.50 (183.75, 256.25)	214.00 (179.00, 250.00)	−0.802	0.422
TC (mmol/L)	4.57 (3.89, 5.26)	4.30 (3.67, 5.02)	−2.488	0.013
TG (mmol/L)	1.74 (1.16, 2.26)	1.57 (1.03, 2.41)	−1.569	0.117
HDL‐C (mmol/L)	1.02 (0.84, 1.15)	1.09 (0.91, 1.27)	−3.495	< 0.001
LDL ‐C(mmol/L)	2.52 (2.00, 3.21)	2.30 (1.84, 2.86)	−3.162	0.002
Serum uric acid (μmol/L)	327.90 (273.30, 390.15)	325.16 (262.10, 383.80)	−1.088	0.276
SCr ≥ 110 (μmol/L)	8 (5.5%)	31 (2.3%)	3.912	0.048
**Echocardiography**				
LVEDD ≥ 50 mm	68 (51.9%)	716 (59.4%)	2.712	0.100
LVEF ≤ 40%	7 (5.3%)	32 (2.7%)	2.144	0.143
**Coronary angiography**				
1 vessel	31 (21.2%)	433 (32.6%)	7.857	0.005
2 vessels	45 (30.8%)	424 (31.9%)	0.071	0.790
3 vessels	70 (47.9%)	472 (35.5%)	8.744	0.003
**Drugs**				
Aspirin	136 (93.2%)	1320 (99.3%)	39.411	< 0.001
Clopidogrel	118 (80.8%)	1043 (78.5%)	0.431	0.512
Ticagrelor	17 (11.6%)	276 (20.8%)	6.879	0.009
β‐blocker	83 (56.8%)	675 (50.8%)	1.933	0.164
ACEI/ARB	67 (45.9%)	598 (45.0%)	0.042	0.837
Statins	135 (92.5%)	1315 (98.9%)	29.385	< 0.001
**Indices**				
CRI‐I ≥ 3.350	137 (93.8%)	960 (72.2%)	32.204	< 0.001
CRI‐II ≥ 1.697	134 (91.8%)	955 (71.9%)	27.023	< 0.001

Abbreviations: ACEI/ARB, angiotensin‐converting enzyme inhibitors/angiotensin II receptor blocker; AMI, acute myocardial infarction; CAD, coronary artery disease; CRI‐I, Castelli's risk index‐I; CRI‐II, Castelli's risk index‐II; HDL‐C, high‐density lipoprotein cholesterol; HF, heart failure; LDL‐C, low‐density lipoprotein cholesterol; LVEDD, left ventricular end‐diastolic diameter; LVEF, left ventricular ejection fraction; MACEs, major adverse cardiovascular events; SCr, serum creatinine; TC, total cholesterol; TG, triglyceride; UA, unstable angina; WBC, white blood cell.

### 3.2 ROC Curve Analysis

We performed a ROC curve analysis to determine the optimal cutoff values of CRI‐I and CRI‐II for predicting MACEs (Table 2). The area under the curve (AUC) for CRI‐I was 0.621 (*p* < 0.001, 95% confidence interval [CI]: 0.579–0.664), and optimal diagnostic cutoff point was 3.350. The AUC for CRI‐II was 0.620 (*p* < 0.001, 95% CI: 0.575–00664), and optimal diagnostic cutoff point was 1.697.

**Table 2:** Receiver operating characteristic (ROC) curve analysis for predictive factors of MACEs.

Variables	AUC	95% CI	*p*‐value	Cutoff
CRI‐I ≥ 3.350	0.621	0.579–0.664	< 0.001	3.350
CRI‐II ≥ 1.697	0.620	0.575–0.664	< 0.001	1.697

Abbreviations: AUC, area under curve; CI, confidence interval; CRI‐I, Castelli's risk indices‐I; CRI‐II, Castelli's risk indices‐II; MACEs, major adverse cardiovascular events.

### 3.3 Cox Regression Analyses

Univariate and multivariate Cox regression analyses were performed to assess the independent predictors of MACEs. The variables that maintained statistical significance (*p *< 0.05) in the univariate Cox regression analysis were included in the multivariate Cox regression analysis. As shown in Table 3, we divided the indices into three groups according to tertiles, and after adjusting for a history of HF and cardiogenic shock, the multivariable Cox regression analysis revealed that the risk of MACEs increased gradually with increasing CRI‐I (hazards ratio (HR) [95% CI]: T1: reference, T2: 2.067 [1.314, 3.249], T3: 2.065 [1.314, 3.244]; p for trend = 0.003) and CRI‐II (HR [95% CI]: T1: reference, T2: 2.002 [1.262–3.176], T3: 2.334 [1.484–3.671]; p for trend = 0.001), which indicated that CRI‐I ≥ 3.350 and CRI‐II ≥ 1.697 were independent predictors of MACEs in patients with ACS undergoing PCI.

**Table 3:** Cox proportional hazards models of the risk of MACEs according to tertiles of the indices.

		Model 1HR (95% CI) *p* value		Model 2HR (95% CI) *p* value	
CRI‐I ≥ 3.350	T1	1 (Reference)	—	1 (Reference)	—
	T2	2.114 (1.345–3.323)	0.001	2.067 (1.314–3.249)	0.002
	T3	2.250 (1.439–3.520)	< 0.001	2.065 (1.314–3.244)	0.002
	P for trend	0.001		0.003	
CRI‐II ≥ 1.697	T1	1 (Reference)	—	1 (Reference)	—
	T2	2.109 (1.330–3.343)	0.001	2.002 (1.262–3.176)	0.003
	T3	2.518 (1.606–3.949)	< 0.001	2.334 (1.484–3.671)	< 0.001
	P for trend	< 0.001		0.001	

*Note:* Model 1: Unadjusted. Model 2: Adjusted for history of HF and cardiogenic shock.

Abbreviations: CI, confidence interval; CRI‐I, Castelli's risk indices‐I; CRI‐II, Castelli's risk indices‐II; HF, heart failure; HR, hazard ratio; MACEs, major adverse cardiovascular events.

### 3.4 Survival Analyses

**Figure 1:**
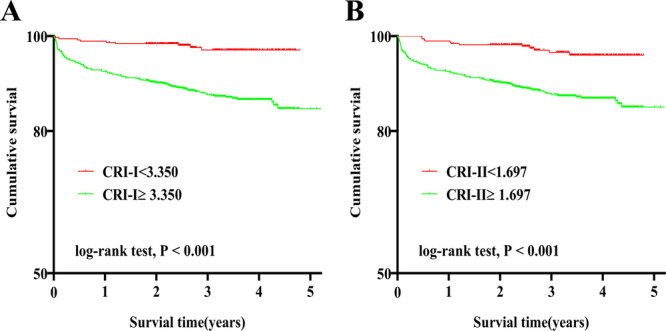
Kaplan–Meier curves for cumulative survival of CRI‐I (A) and CRI‐II (B) The Kaplan–Meier curve was plotted using the survival data collected at the follow‐up timepoints to compare different survival rates in two groups. CRI‐I, Castelli's risk index‐I; CRI‐II, Castelli's risk index‐II.

The Kaplan–Meier curve was plotted using the survival data collected at the follow‐up timepoints to compare different survival rates in different groups. Figure 1 shows that the cumulative survival rate in the CRI‐I ≥ 3.350 and CRI‐II ≥ 1.697 groups was significantly lower than that in the CRI‐I < 3.350 and CRI‐II < 1.697 groups, respectively (log‐rank tests: all *p* < 0.001).

### 3.5 Restricted Cubic Spline

**Figure 2:**
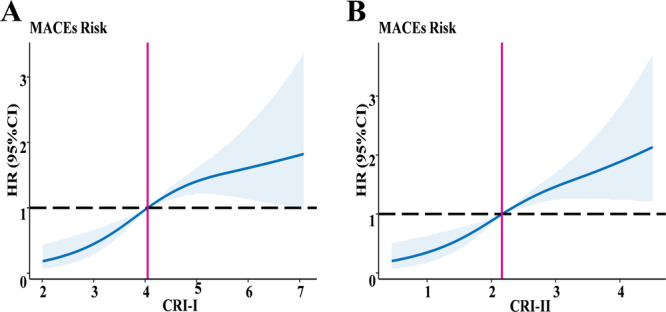
Restricted cubic spline (RCS) of CRI‐I (A) and CRI‐II (B) The RCS was used to visualize the association between the two indices and risk of MACEs. HR = 1 as a reference line. CRI‐I, Castelli's risk index‐I; CRI‐II, Castelli's risk index‐II; HR, hazard ratio; MACEs, major adverse cardiovascular events.

The relationships of the tertiles of CRI‐I and CRI‐II with MACEs are shown in Table 3. The risk of MACEs increased gradually with increasing CRI‐I and CRI‐II tertiles. The RCS was used to visualize the association between the two indices and risk of MACEs (Figure 2). The values of CRI‐I and CRI‐II with HR values close to 1 were 4.048 and 2.164, respectively. Therefore, CRI‐I ≥ 4.048 and CRI‐II ≥ 2.164 were independent risk factors for MACEs. A linear relationship was observed between MACEs and CRI‐I (P_overall_ < 0.001, P_non‐linearity_ = 0.206) and CRI‐II (P_overall_ < 0.001, P_non‐linearity_ = 0.066).

### 3.6 Development and Validation of the Nomogram

**Figure 3:**
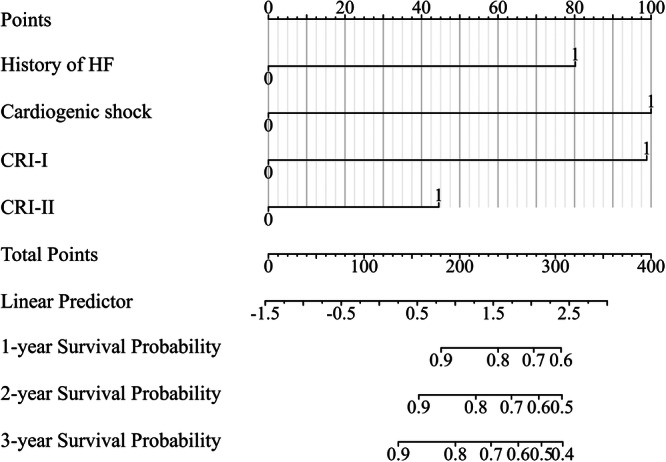
Nomogram to estimate the risk of MACEs in patients with ACS undergoing PCI. The value of each variable was assigned a score on the point scale axis, and by adding the corresponding score for each variable, a total score could be calculated and projected to the 3‐year survival probability. ACS, acute coronary syndrome; CRI‐I, Castelli's risk index‐I; CRI‐II, Castelli's risk index‐II; HF, heart failure; PCI, percutaneous coronary intervention.

**Figure 4:**
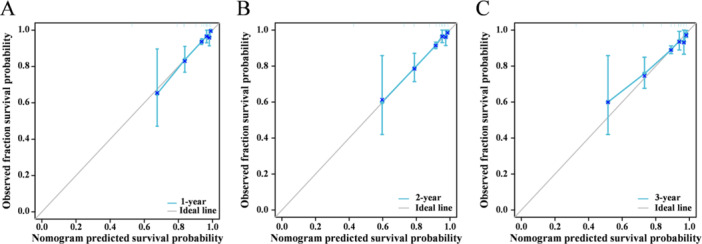
(A–C) Calibration plots of predicted 1‐, 2‐, and 3‐year MACEs. The predicted curve (gray) was close to the actual curve (blue), indicating the calibration curves for the probability of survival demonstrated good agreement with the actual observations. MACEs, major adverse cardiovascular events.

After the univariate analysis, the following variables were entered into the multivariate Cox regression analysis: age ≥ 65 years, UA, AMI, dyslipidemia, history of HF, cardiogenic shock, SCr ≥ 110 (μmol/L), CRI‐I ≥ 3.350, and CRI‐II ≥ 1.697. The multivariate analyses showed that the occurrence of MACEs was significantly correlated with a history of HF, cardiogenic shock, CRI‐I ≥ 3.350, and CRI‐II ≥ 1.697; therefore, we identified history of HF, cardiogenic shock, CRI‐I ≥ 3.350, and CRI‐II ≥ 1.697 as independent predictors of MACEs in patients with ACS undergoing PCI. The prognostic nomogram model was constructed based on the above multivariate analysis results (Figure 3). The value of each variable was assigned a score on the point scale axis, and by adding the corresponding score for each variable, a total score could be calculated and projected to the 3‐year survival probability. A higher total score indicated a lower 3‐year survival probability. The internal validation of the nomogram was conducted using the training set. The calibration curve showed that the predicted curve was close to the actual curve, thus indicating that the calibration curves for the probability of survival at 1, 2, and 3 years demonstrated good agreement with the actual observations (Figure 4).

## 4 Discussion

As we knowledge, this is the first study that used a nomogram based on the CRI to predict prognosis in patients with ACS undergoing PCI. The main findings can be summarized as follows. First, CRI‐I ≥ 3.350 and CRI‐II ≥ 1.697 were associated with the prognosis, and the risk of MACEs increased gradually with increasing CRI‐I and CRI‐II tertiles. Second, CRI‐I ≥ 3.350 and CRI‐II ≥ 1.697 were independent predictors of MACEs in patients with ACS undergoing PCI. Third, the nomogram demonstrated good predictive performance of survival probability for 3 years confirmed by the calibration curve.

Atherosclerosis is a major driver of MACEs and the death in patients with nonalcoholic fatty liver disease [17, 18]. Various physiological and pathological processes are involved in the pathogenesis of atherosclerosis, including inflammatory reaction, oxidative stress, dysregulation of lipid metabolism, and improper immune responses [19, 20, 21]. Among lipid parameters, total cholesterol demonstrates superior predictive value for carotid intima‐media thickness and the presence of carotid plaque than other lipid parameters [22]. One research shows that the serum concentration of TC is the most important risk factor for ischemic heart disease in men [23]. HDL‐C exerts antiatherogenic effects by removing cellular lipids and reversing cholesterol transport [24, 25]. Furthermore, HDL‐C levels are negatively associated with the risk of coronary heart disease and could potentially reduce the morbidity of CVD [26, 27]. LDL‐C is one of the most crucial validated targets in clinical medicine, and a large body of evidence has established a significant link between LDL‐C levels and CVD [28, 29]. Based on the mechanisms and functions of TC, HDL‐C, and LDL‐C in CVD, the CRIs, which combine TC, HDL‐C, and LDL‐C, have been found to be associated with different diseases. Acar et al. evaluated proatherogenic lipid profiles in patients with Behçet's disease and they demonstrated that significantly higher level of CRI‐I and CRI‐II were observed in case group [30]. Albuquerque et al. emphasized that CRI‐I and CRI‐II are important lipid markers for managing CVD risk in patients with multiple sclerosis [31]. In a recent study, after comparing the predictive efficacy of different atherogenic indices for metabolic syndrome in patients with schizophrenia, the author suggested that higher CRI‐I and CRI‐II levels are associated with metabolic syndrome [32]. In addition, Klisic et al. found that CRI‐I and CRI‐II are closely and independently related to nonalcoholic fatty liver disease status [33]. However, there have been no studies on the prognostic value of CRI‐I and CRI‐II in patients with ACS undergoing PCI. Unlike single index, such as lipoprotein(a) and high sensitivity c‐reactive protein, CRI‐I and CRI‐II integrate three dimensions of atherogenesis, which improved the predictive performance of diseases. The results of a retrospective study showed that CRI‐I has higher predictive value of in‐hospital mortality of patients with COVID‐19 compared with MHR, LDL‐C, TC and Non‐HDL‐C [34]. CRI‐II was independent predictor of coronary slow flow, and had higher AUC than AIP [10].

In our study, we combined TC, HDL‐C, and LDL‐C to predict the prognosis of patients with ACS who underwent PCI. The performance of CRI‐I and CRI‐II, as novel indices, was assessed between the MACEs and non‐MACEs groups. After adjusting for various confounders, the multivariate Cox regression models showed that CRI‐I and CRI‐II were correlated with poor prognosis and were independent risk factors for MACEs in patients with ACS undergoing PCI. The RCS confirmed the linear trends between CRI‐I, CRI‐II, and MACEs. Nomograms, as a visualization tool, are more accurate than conventional markers for predicting the prognosis of diseases, and our nomogram exhibited better clinical value in predicting 1‐, 2‐, and 3‐year survival. In this study, we utilized composite lipid indices with the aim of enhancing the prediction of prognosis risk, thereby providing a more comprehensive and accurate assessment.

This study has several limitations. First, this was a single‐center study, and selection bias may have occurred. Second, CRI index and covariates we collected only at baseline, this single‐time‐point measurement lack of the dynamic surveillance, therefore, future studies are needed to explore the dynamic changes of CRI index and provide more valuable prediction on cardiovascular diseases. Third, although multivariate Cox models were employed to control confounding factors, unmeasured confounding remains a possibility. Finally, a key limitaion is the absence of external validation, in subsequent studies, we will collaborate multiple centers to collect more external cohort data to validate the stability of our research.

## 5 Conclusions

Our study demonstrated that CRI‐I ≥ 3.350 and CRI‐II ≥ 1.697 were associated with prognosis and may serve as independent predictors of MACEs in patients with ACS undergoing PCI, which may help identify individuals with high risk of MACEs and guide effective treatment modalities in ACS patients.

## Author Contributions

**Yan Liu:** conceptualization, formal analysis, methodology, software, visualization, writing – original draft, writing – review and editing. **Ge Song:** methodology. **Ying Zhang:** resources. **Weichao Shan:** data curation. **Yuewen Qi:** data curation. **Xinchen Wang:** methodology. **Chen Wei:** methodology. **Jingyi Liu:** data curation. **Lixian Sun:** conceptualization, methodology, supervision, validation, writing – original draft, writing – review and editing.

## Ethics Statement

This study was approved by the Ethics Committee of the Affiliated Hospital of Chengde Medical University (Number: CYFY LL2015006). All participants provided written informed consent.

## Conflicts of Interest

The authors declare no conflicts of interest.

## Data Availability

The raw data supporting the conclusions of this article will be made available by the authors without undue reservation.
